# Potential of Honeycomb-Filled Composite Structure in Composite Cross-Arm Component: A Review on Recent Progress and Its Mechanical Properties

**DOI:** 10.3390/polym13081341

**Published:** 2021-04-20

**Authors:** Abd Latif Amir, Mohamad Ridzwan Ishak, Noorfaizal Yidris, Mohamed Yusoff Mohd Zuhri, Muhammad Rizal Muhammad Asyraf

**Affiliations:** 1 Department of Aerospace Engineering, Universiti Putra Malaysia, 43400 UPM Serdang, Selangor, Malaysia; nyidris@upm.edu.my (N.Y.); asyrafriz96@gmail.com (M.R.M.A.); 2 Aerospace Malaysia Research Centre (AMRC), Universiti Putra Malaysia, 43400 UPM Serdang, Selangor, Malaysia; 3 Laboratory of Biocomposite Technology, Institute of Tropical Forestry and Forest Products (INTROP), Universiti Putra Malaysia, 43400 UPM Serdang, Selangor, Malaysia; 4 Advanced Engineering Materials and Composites Research Centre (AEMC), Department of Mechanical and Manufacturing Engineering, Universiti Putra Malaysia, 43400 UPM Serdang, Selangor, Malaysia; zuhri@upm.edu.my

**Keywords:** PGFRP composites, cross-arms, transmission towers, honeycomb filled, sandwich structures

## Abstract

Nowadays, pultruded glass fiber-reinforced polymer composite (PGFRPC) structures have been used widely for cross-arms in high transmission towers. These composite structures have replaced cross-arms of conventional materials like wood due to several factors, such as better strength, superior resistance to environmental degradation, reduced weight, and comparatively cheaper maintenance. However, lately, several performance failures have been found on existing cross-arm members, caused by moisture, temperature changes in the atmosphere, and other environmental factors, which may lead to a complete failure or reduced service life. As a potential solution for this problem, enhancing PGFRPC with honeycomb-filled composite structures will become a possible alternative that can sustain a longer service life compared to that of existing cross-arms. This is due to the new composite structures’ superior performance under mechanical duress in providing better stiffness, excellence in flexural characteristics, good energy absorption, and increased load-carrying capacity. Although there has been a lack of previous research done on the enhancement of existing composite cross-arms in applications for high transmission towers, several studies on the enhancement of hollow beams and tubes have been done. This paper provides a state-of-the-art review study on the mechanical efficiency of both PGFRPC structures and honeycomb-filled composite sandwich structures in experimental and analytical terms.

## 1. Introduction

When power transmission lines were first introduced in Malaysia, wooden cross-arms were used on transmission towers [1]. In 1929, *Chengal* wood (*Neobalanocarpus*) was used for the wooden cross-arms on 66 kV towers, and was further expended and applied for 132 kV suspension towers in 1963 [2]. After a certain period of time, it was found that the wooden cross-arms started to fail due to aging activities, and by the late 1990s, matured *Chengal* woods were not easily obtained to make good quality cross-arms. In addition, a defective wooden cross-arm due to natural wood defect was found after 14 years of service, which was less then estimated service period [3,4,5,6]. These problems led to the search for alternative materials to substitute wooden cross-arms. Therefore, the pultruded glass fiber-reinforced polymer composite (PGFRPC) cross-arms were installed on the top, middle, and bottom phases of selected towers as the first pilot project in Malaysia [7,8]. The pultruded manufacturing method of PGFRPC was chosen because composite structures were easily mass produced and exhibited excellent characteristics compared to wooden cross-arms [9,10,11].

Nowadays, even though the failure of cross-arm structures has become a critical issue, there have been insufficient ideas on how to overcome this. At present, issues pertaining to the sustainability of existing PGFRPC cross-arm members are being addressed by temporarily strengthening the materials with additional structural members. Mohamad et al. [12] studied numerous structural deformation behaviors for improving cross-arms and came up with the use of sleeve installations on both arms. In addition, other fields are studied. For example, in the field of construction, sleeve installations are widely used to join and strengthen the connection between beams and columns or other connections due to their good performance [13,14]. Another method of improvement done by previous researchers was by installing bracing on cross-arm members. From numerical simulations, some researchers have used bracing support on cross-arm members, and subjected one end of the cross-arm to a specific load [15]. Hussein et al. introduced a different conceptual design for installing bracing, which is suitable for a 3-cross-arm member type [16]. Furthermore, the use of bracing has also been applied for designing the cross-arm tower of transmission lines. However, the bracing system can contribute to slip potentials due to the large stress placed on the beams.

There is still a lack of research that focuses on how to overcome cross-arm failures. However, several studies have applied a filled structure into a hollow beam or tube; this method has been increasing among researchers since the idea can also be applied to improve the cross-arm structures [17,18]. The concept of filling structures mostly employ either a polymeric foam core [19,20,21], wood core [22,23], or honeycomb core as a verified filler for hollow beams and tubes [24,25,26]. Qin et al. investigated the low-velocity impact response of a fully clamped corrugated sandwich beam with a metal foam-filled folded plate core through analytical and numerical simulations. The yield criteria, dynamic, and quasi-static solutions were determined by the large deflection of the fully clamped metal foam-filled corrugated sandwich beams. Both results of finite element simulations and quasi static predictions agreed well with the use of the circumscribing and inscribing yield criteria [19,27].

Another research on honeycomb-filled aluminum square tubes was performed by Zarei et al. in terms of finding a more efficient and lighter crash absorber. The axial and oblique impact crash tests on empty and honeycomb-filled aluminum square tubes were compared [26]. The study showed that the selection of an appropriate honeycomb density and tube/beam thickness must be critically considered. In addition, a structure would lose its weight when the density of filler is higher than the critical filler density [28,29]. A series of experimental tests and numerical simulations were performed to demonstrate the strengthening effect of the honeycomb design in honeycomb-filled tubes. Honeycombs with different densities and tubes with different thicknesses were selected [30,31]. A higher strengthening effect occurred in the case of a high density honeycomb, but its weight efficiency was lost by the structure. It was demonstrated that an optimal honeycomb-tube combination must be selected for an effective performance in a light honeycomb-filled crash absorber [24,25].

Since cross-arm structures obey the principle of the cantilever beam structure, several mechanical behaviors on honeycomb-filled structures need to be analyzed, such as load-carrying capacity, flexural, creep, and failure mood behavior. All these studies should be conducted to suggest possible material changes in the structural design of the cross-arm system, for the purpose of limiting or preventing failures. Nonetheless, fundamental studies to propose a new material design for potential use in these members remain fairly unexplored. Hence, the potential of using new filling structures in this scope will be studied in this work.

## 2. Recent Progress and Applications of Composite Materials

Many applications have been developed by scientists and designers in parallel with this view to create lightweight products with cheaper costs and promising mechanical efficiency. This section focuses on the literature review of works by researchers in the current development of fiber-reinforced polymer composite materials and products [32]. Polymer composite materials are very important in various products and applications in some sectors, such as the automotive industry, mechanical industry [33,34], marine industry, aerospace industry [35,36], civil industry, and the infrastructure industry [37,38]. In terms of ensuring environmentally friendly and sustainable technology growth, some industries have adopted natural polymeric composite materials in their applications [39,40]. Table 1 shows several fiber-reinforced polymer composite applications and properties in different areas.

Various fiber composite materials, integrated with the combination of distinct base materials and manufacturing techniques, provide higher material properties compared to the use of pure materials, polymers, or alloys; this makes fiber-reinforced polymer (FRP) composites suitable for desired application [58,59]. Besides that, the FRP composites also have the advantage of being able to replace most of the conventional composite materials [60]. As a reinforcement in a composite structure matrix, fibers act as a load-carrying element. While the matrix material holds fibers in the required location and orientation, it also facilitates stress transfer and protection against the environment. For a variety of applications, where greater strength to weight ratio is needed, FRP materials have been found to be superior to metals in applications involving composite materials [61,62].

## 3. Manufacturing Processes of Composite Products

In this century, composite materials have been found to the most promising materials in many applications. Presently, high demands of composite-reinforced polymers available in the markets, either of synthetic fibers or natural fibers, are manufactured due to their higher strength and lightweight properties [63,64] for specific applications. However, the performance of composite materials predominantly depends on their constituent elements and manufacturing techniques [65]. In general, different techniques are used to produce polymer composite products, but not all of them are suitable for large-scale output productions. For example, hand layout techniques are useful for custom-shaped goods with low production rates, but are less cost-effective in the manufacturing of composite materials [66,67,68]. On the other hand, the introduction of injection molding is extremely advantageous for the manufacturing of composite materials in large quantities of components, though the manufacturing cost is incurred by the customization of these components [69]. The highest quality of composite manufacturing production strategies are possible due to the progress in automation [70].

**Figure 1:**
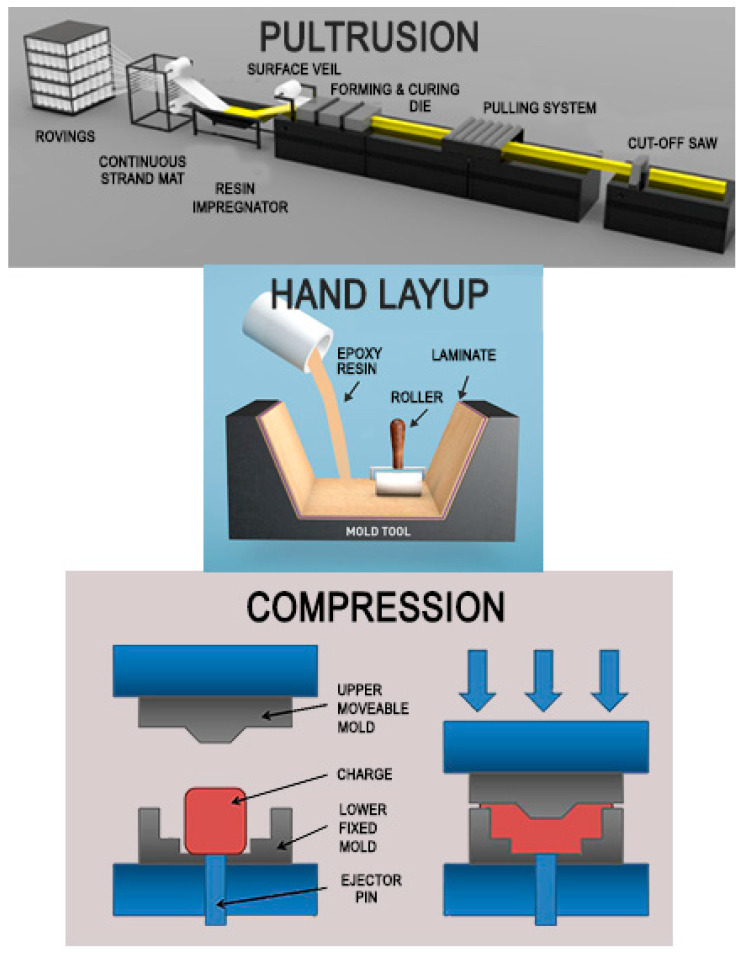
Various manufacturing processes of polymeric composite products.

Resin transfer molding, compression molding, hot pressing, injection molding, vacuum infusion, filament winding, hand and spray lay-up methods [71] are the majority of manufacturing techniques currently is use to produce polymeric composite products. Figure 1 provides examples of some manufacturing processes in a schematic diagram [65,72]. In some cases, in contrast to synthetic fiber-reinforced polymer composites, the application of natural fiber-reinforced polymer composites normally reveal certain problems, such as low stability and compliance with the use of these manufacturing techniques [73,74,75]. Therefore, performing a pre-treatment process of fibers before implementing the manufacturing process will become the best method to further enhance manufacturability [76].

The hand lay-up method is the oldest method used in the manufacturing process to obtain composite materials [77]. The first step in the hand lay-up method begins by placing glass fiber materials manually inside a mold; then, the matrix materials are spread evenly over the fiber layers. Lastly, entrapped air is removed with squeegees and rollers [72]. This technique has been a key part in the composite manufacturing industries due to its ability to create complex shapes, to maintain a reasonable low cost, to provide adaptability and potentially to allow short lead times [78].

For the spray lay-up method, the process implements a handgun to spray the chopped fibers and resins concurrently onto the subjected mold. Later, rollers or squeegees are applied to the fiber-resin layers to fuse the composite laminates evenly [79]. This technique is considered as an open mold technique; the chopped fibers provide good conformability and this method is quite faster than the hand lay-up method [80]. However, the spray lay-up method is highly dependent on the experience of the operator in achieving an acceptable degree of uniformity [81].

Other than that, the vacuum infusion method for composite products particularly uses the principle of pressure as implemented in resin transfer molding and vacuum bagging [82]. In this method, the fiber material is placed on a prepared release agent or film; then, this structure is covered with a peel ply to ensure that the vacuum bags of film and resin channels can be separated after infusion to cure the resin [82]. The advantages of the vacuum infusion technique are its low cost and the good quality of its final products. However, the vacuum bags used in this infusion technique are not reusable after the processing cycle, so high amounts of waste are generate [83,84,85].

Another common method in fabricating composite material products is the compression method, which requires the prepared reinforcement package to be placed in between two halves of the mold, namely the cavity mold (lower fixed) and the form mold (upper movable). Then, the package is pressed against each mold halves to get the desired shape of the mold [65,86]. This method fabricates a product with high strength and good dimensional stability, especially in automotive and appliance applications [87]. However, in the compression method, the fibers are discontinuous and their alignment depends on the melt flow during the manufacturing process [88].

For injection molding, the manufacturing process could facilitate the rapid production of composite products with intricate and complex geometry parts [89]. This method is implemented widely in aerospace, automotive, and biomedical products and sectors [90,91]. In the injection molding process, the composite compositions, including fiber and matrix, are fed through a hopper and then conveyed by a screw with a heated barrel. Once the required amount of material is melted in the barrel, the screw injects the material through a nozzle into the desired shape mold [92].

**Figure 2:**
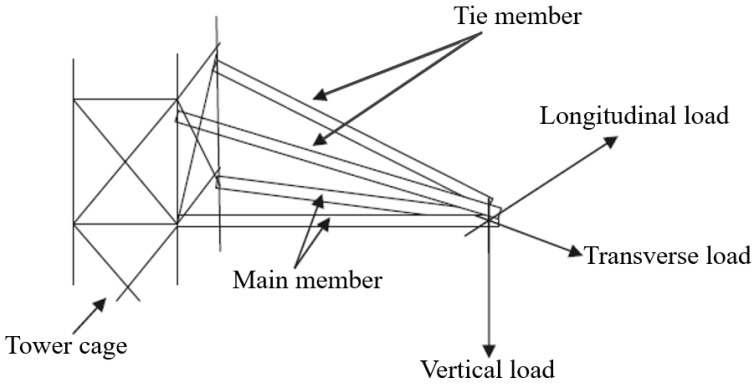
Schematic diagram of cross arm assembly in transmission tower [11].

Lastly, pultrusion is another method used to produce long, symmetrical, and consistent profile products such as pipes [93]. The pultrusion process involves pulling the reinforced fibers through a thermoset resin bath for impregnation. Afterward, the reinforcement is allowed to enter into a heated forming die, as shown in Figure 2. Later, the composite products are shaped at the die cavity and curves [94]. Finally, the consolidated part is pulled by a continuous pulling system and then cut into segments of desired lengths [95]. The pultruded composite products have many advantages, including lightness, high strength and stiffness, easy and rapid installation, anti-corrosion, and acoustic insulation [96]. The pultrusion method for GFRP composites is being used increasingly as a replacement for conventional materials in various civil and mechanical engineering structures, such as framed building, beams, cross-arms, and bridges [97,98,99]. All in all, it is worth to note that the cross-arm beams are most suitable to be fabricated and manufactured using the pultrusion process since it has symmetrical square hollows and long shapes [15]. Moreover, the pultrusion process can produce a rapid rate of production with a good surface finish and efficient mechanical performance [98].

## 4. Transmission Line Systems in Malaysia: Latticed Transmission Tower

In the past several decades, many development concepts of overhead power line transmission tower designs have emerged, mainly resultant of the growing environmental constraints despite the increase in demands of power. In general, the transmission tower design must counter the weight of the transmission conductor at a certain height from the ground [100]. Cross-arm is one of the main structures on the transmission tower, which supports the transmission conductor. Lately, the PGFRPC has been chosen in the development of cross-arms for high transmission towers, especially in Malaysia [99]. Essentially, their advantages include high strength, corrosion resistance, chemical stability, and good performance as insulators in lightning impulse strength for cross-arms in high transmission towers [11]. Three main electrical cables currently implemented in the Malaysian power grid system of transmission lines are 132 kV, 275 kV, and 500 kV cables [99,101]. The largest transmission structure developed in Malaysia is the 500 kV, followed by the 275 kV large transmission line system, and the 132 kV medium transmission system [102]. Figure 2 displays the schematic diagram of cross-arm assembly on transmission towers.

### 4.1. PGFRPC Cross-Arm in Latticed Transmission Tower

Since 1929, the 132 kV cross-arm assembly has been commissioned in the Malaysian electrical grid, using *Chengal* wood as the structural material [103]. Since it has better mechanical efficiency and excellent quenching of the electric arc [104,105], the wooden cross-arm was selected to be mounted on transmission towers. In addition, wood is a resourceful material for purposeful structures and has been used for a long time. The wood-based materials are also readily available with low cost processing for large quantities. The old wooden timber cross-arm typically needs further inspection over a long period of operation, as it would be subjected to attacks by natural bio-degradation such as rotting, and natural adversaries such as woodpeckers and termites [106,107]. This has raised several issues with respect to the wooden cross-arm, in particular, the structural failure which appears to occur after 20 years of service on transmission towers [108]. Moreover, apart from natural wood defects [106,109,110,111], the structural deficiencies of the cross-arm on transmission towers are typically due to creep deformation in long-term operations. In certain situations, the failure of the cross-arm on the transmission tower would disrupt end users’ electrical supply and would also result in higher maintenance costs for the electrical company [99]. Furthermore, the failed cross-arm structure may also result in higher chances of causing casualties to nearby pedestrians [112].

**Figure 3:**
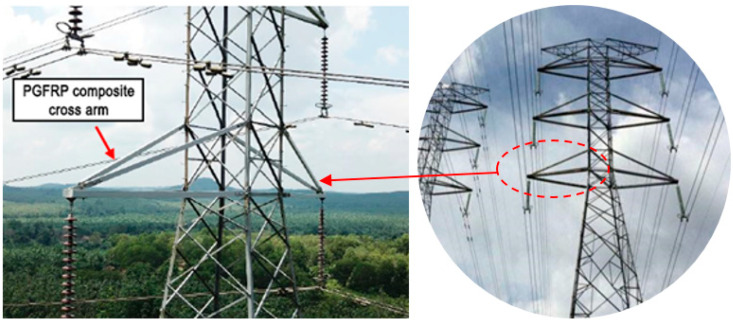
Dimension of latticed transmission tower.

The researchers were urged by this troubling situation to replace the cross-arms with new materials, such as polymeric composites. Pultruded glass fiber-reinforced polymer (PGFRP) composite cross-arms have been implemented (as shown in Figure 3) on transmission towers to replace the existing wooden cross-arms [113] to fulfil the criteria of seeking alternatives. The benefits of using PGFRP composites are due to their being lightweight, not bio-degradable, and versatile in nature [114]. Daniel et al. demonstrated a linear viscoelastic model combining the elastic behavior of fiber and the viscoelastic nature of matrix to represent the long-term behavior of PGFRP composite materials [115]. Other researchers have studied the mechanical performance of PGFRP composites subjected to long-term environmental effects, including water/moisture, and low/high temperature [95]. However, there is still a lack of PGFRP composite cross-arm studies on full-scale mechanical efficiency, structural stability, and integrity to withstand long-term duty loads [116]. Most studies and research works are currently focused on computer simulations, coupon-scale (small) experiments, and beam analysis, which do not characterize the assembly holistically.

### 4.2. PGFRPC Cross-Arm: Current Issues and Problems

**Figure 4:**
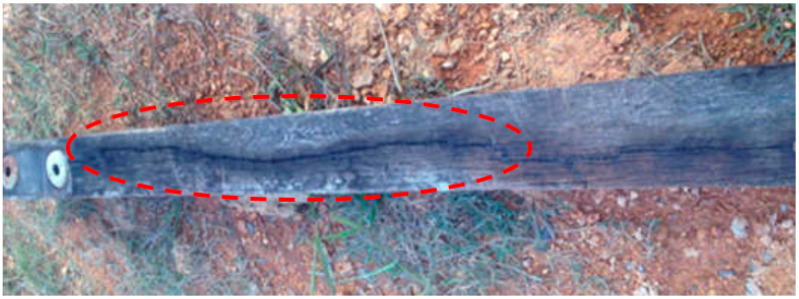
Wooden cross arm failure due to lightning strike.

Lightning performance is one of the main considerations in the development of cross-arms for high transmission towers [117]. In the early stages of cross-arm development, some researchers studied on lightning performance on cross-arms made of wood and steel [118]. As technology progresses, the development of cross-arms also changes. The PGFRPC are used for cross-arms in the application of high transmission towers. By using experimental and simulation works, Rawi et al. [1] showed that PGFRPC cross-arms have a higher dielectric strength compared to wooden cross-arms in terms of performance against lightning strikes, as shown in Figure 4. Other researchers tried to overcome the lightning performance of transmission lines by developing grounding systems for transmission line towers [119,120].

As the main objective of cross-arm design must counter the weight of the transmission conductor at a certain height from the ground, most researchers have struggled to come up with improvements of the cross-arm design [121,122,123]. For instance, Munusamy et al. [11] studied the mechanical behavior of composite cross-arms made of pultruded rods with crimped metallic end clamps design. This study encourages the possibility of using composite cross-arms with a FRP tower body to reduce the horizontal phase distance, i.e., building more compact transmission lines. In addition, Hussein et al. [16] introduced a conceptual design by applying a bracing on the main and tie cross-arm members to achieve the optimal solution in the development of transmission tower cross-arms. Selvaraj et al. [124] discussed experimental studies on linear-elastic responses, including buckling consideration for X-braced panel design of transmission line towers made of FRP pultruded sections, as replacements for rolled steel angle sections.

A number of researchers have focused on designing improvements for high transmission tower cross-arms by using the same material, i.e., PGFRPC. Table 2 explains the current research works for implementing PGFRPC cross-arms.

**Figure 5:**
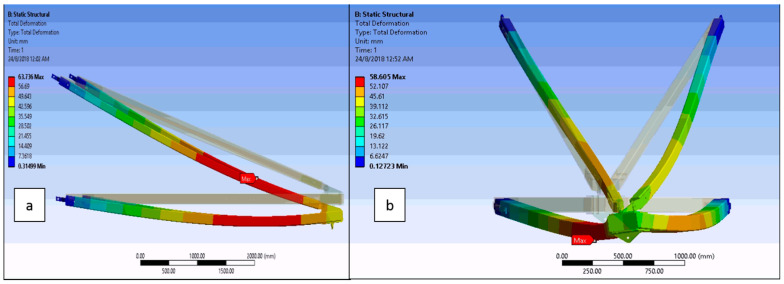
Schematic diagram of cross-arm structure when exposed applied force at the end of the cross-arm structure (**a**) side view and (**b**) front view [127].

Many computer simulations have been carried out by several researchers to characterize the mechanical properties of PGFRP cross-arms. Those research works have been executed on composite cross-arms, which include determining the effects of laminate properties on cross-arm failures [125], the effects of laminate stacking sequence on cross-arm performance [126], and the effects of static loading with various configurations on cross-arm behavior [15]. Figure 5 illustrates the examples of failure, such as the simulated deformation results of the 275 kV cross-arm on transmission towers in normal and broken wire conditions [127].

### 4.3. Critical Failure Issues of Cross-Arm

There are many computer simulations carried out by several researchers to characterized the mechanical properties of PGFRP cross arm. Those research works are executed on composite cross arm which includes effect of laminate properties on cross arm’s failure [125], the impact of laminate stacking sequence on cross arm’s performance [126] as well as the effect of static loading with various configurations on cross arm behaviors [15]. Figure 5 illustrates the failure examples, the simulated deformation results of 275 kV cross arm in transmission tower in normal and broken wire conditions [127].

**Figure 6:**
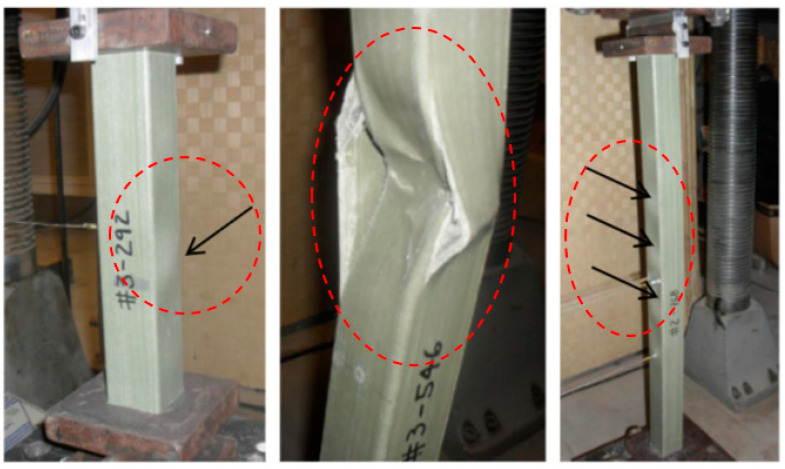
Failure observations (buckling) experienced by pultruded glass fiber-reinforced polymer composite (PGFRPC) square hollow tube.

From an experimental point of view, various research activities have been performed to analyze the cross-arm beams and structures on latticed towers. A study on PGFRP beam failure was carried out by Cardoso et al. [129] with regard to developing a comprehensive equation for PGFRP square tube columns under a concentric compression. The study revealed that a post-buckling effect has been observed on the properties and interaction between crushing, local, and global buckling, as depicted in Figure 6. Moreover, Selvaraj et al. [11] also conducted a mechanical performance test for composite cross-arms and validated the results with finite element (FE) analysis to study the cross-arm failure.

In this manner, the square hollow section of PGFRPC beams are highly potential to buckle and fail when subjected to a high concentration load. It is necessary to incorporate a honeycomb-filled sandwich structure to allow for a higher mechanical performance in resisting buckling due to the long-term loading of electrical cables and insulators. In the next subsection, a review on mechanical behavior of filled sandwich composites structures are elaborated.

## 5. Overview of Composite-Filled Structures

**Figure 7:**
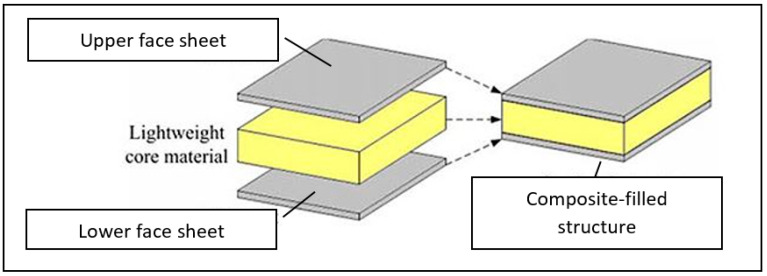
Sandwich composite structure layout.

In recent years, composite-filled structures have rapidly gained great attention as a new approach, due to their excellence in flexural characteristics, creep behavior, stiffness, energy absorption, and load-carrying capacity. Basic principles of composite-filled structure is composed of face sheet and core material, in which the core material is designed in between the face sheet, as shown in Figure 7.

**Figure 8:**
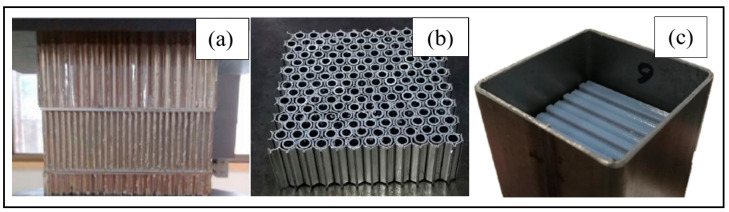
Innovation of honeycomb structures. (**a**) Tandem honeycomb structure, (**b**) honeycomb structure, and (**c**) honeycomb filled structure.

These core designs can be designed in numerous forms to develop the sandwich structure, such as honeycomb, balsa wood, foam, corrugated, tetrahedral truss, and various bio-inspired cores [23,130,131,132,133]. Figure 8 displays the innovation of composite-filled structure applying honeycomb core to produce strong, stiff and lighter weight product [134,135,136]. In general, the face sheets are thinner as compared to the core which allow the material to be strong and stiff with lightweight property. The sandwich structure materials are widely choose depend on the function and application of the structure like lifetime loading, availability and costing [137,138].

The tandem honeycomb structures have been rarely used in study by researchers which less literature involved indirectly to the tandem honeycomb filled structure itself [139]. An investigation on dynamic impact crushing behavior of pyramid multi-layer honeycomb sandwich panels was found in literature [140]. It was found that the energy absorption performance of the pyramid type was better than the uniform type in both efficiency and capacity. The comprehensive experimental study of tandem hexagonal honeycomb structures subjected to axial compression was observed in Wang et al. [136] studies. In this study, the tandem honeycomb structure performed a better mechanical behavior compare to single honeycomb block in term of stable rectangular-like force-compression which has no initial peak force appeared. Although that, the tandem honeycomb structure deformation behavior and mechanical properties is uncertain since their strength and stiffness of each segments are unified [141,142].

Differing from tandem honeycomb-filled structure, another way has been found by embedding the honeycomb cells with foam, tubes or even other polymer materials [143]. The embedded honeycomb structure design can be seen as a container instead of filler. For example, a theoretical model for determination of the mean crushing strength of the foam filled metal hexagonal honeycomb under quasi-static loading was study by Mahmoudabadi et al. [144]. It was found that the mean crushing stress of the foam filled honeycomb structure get the maximum utility rate of space compare to non-embedded structure which lead the structure behave more likely cellular solids. Others have used circular tubes as a honeycomb filler to make an embedding honeycomb structure [145,146]. All the studies focused on energy-absorbing properties of the embedded honeycomb structure itself. Besides that, the required filling pattern still needs more study for the embedded honeycomb structure. In the meantime, the problems of developing a matching relationship and achieving a controllable mode of deformation are still challenging.

The most commonly used in composites filler structure are honeycomb-filled structure due to their merits, simple configuration and easy to manufacture. The used of honeycomb filled structure have been verified as a good filler for circular and square tubes [26,147]. Zhu et al. [148] introduced a new structure comprises of a functionally gradient honeycomb filler and a functionally graded thickness tube namely as double functionally graded (DFG) structure. By comparing the crashing behavior of different structure, this structure exhibits superior energy absorption capacity over other configurations. Compared to traditional tubes, the rise in peak force at the initial stage and the promotion at the plateau stage are easily observed in these types of honeycomb-filled structures. Even, for the densification stage, less displacement is needed. In other words, because of the honeycomb filler, the energy absorption and load carrying capability are certainly improved [143]. It should, however, be noted that there is an obvious matching effect between the filler and container for this type of filling structures [149], and different mechanical responses can be observed through different matching relationships, which are highly dependent on the dominant position. Other related factor of matching effect is the material properties between these two key components. Different type of honeycomb core and different containers used will show different deformation mode [26,149,150].

In term of applications, the composite-filled structure usually involves several hybrid composite materials which are composed of more than one fiber. The most common fiber used in a composite-filled structure is glass fiber, due to its affordable cost. The glass fiber would be combined with other expensive fibers, such as graphite or carbon, to manifest their desirable behaviors which include low density, high specific modulus, and high specific strength. At the end of the product, the hybrid composite-filled structure would significantly improve their mechanical and physical performances compared to individual fiber-based composites. The process involving hybrid compositions has made it possible to produce and attain high composite system materials for different applications.

Various research works have been conducted to investigate the mechanical properties of hybrid composite-filled structures. According to Swolfs et al. [151], a hybrid glass-carbon fibers composite-filled structure has been discovered to allow an increase in strength and energy absorbing characteristics. In recent years, studies on energy absorbing characteristics of hybrid composites have emerged to become major researches on composite-filled structures. For instance, a study was carried out on the effects of sequencing in hybrid layers and on energy absorption for both blunt and hemispherical projectiles of composite-filled structures [152]. A similar behavior was also observed in Muhi et al.’s [153] experimental studies.

In addition, Ashraf et al. [154] evaluated a hybrid composite-filled structure by implementing two kinds of structure and material, such as woven and knitted glass fiber and aramid yarn. Different stacking sequences of fabric plies were studied. They discovered that more energy would be absorbed in the knitted reinforced composite-filled structure as compared to the others. Table 3 lists other related studies and their outcomes.

## 6. Evaluation of Composite-Filled Structures Behavior

Filled composite structure are vital potential prospect for enhancement of PGFRPC cross arm beams in transmission line tower based on finding on this review. The innovation of honeycomb-filled composite structure was highlighted in this review due to their great potential to produce strong, stiff, and lighter weight product. Although PGFRPC cross arm are made up of pultrusion and in square hollow shape, the cross-section geometry in the cross arm beams has caused a very complex behavior as it is subjected to multiaxis force [116]. Hence, it is important to evaluate the behavior of potential honeycomb filled structure so that it can fulfil the mechanical behavior standards as the previous PGFRPC cross arm. This subtopic is focused on recent studies related to the test evaluation for honeycomb-filled composite structures behavior.

### 6.1. Flexural Stiffness Behavior

Most characterization processes of materials or structures need flexural behavior information because it provides the relevant information on the suitability of the design and how the materials perform in real applications. Three-point bending tests were carried out by Xingyu et al. [158] on honeycomb sandwich beams to gain insights into the role of physical dimensions for tuning the flexural property of the sandwich structure. Meanwhile, Vitale et al. [159] studied flexural properties, which focused on bending properties and failure modes of natural and synthetic fiber-reinforced composite sandwich beams. This study carried out three point bending test to validate the mechanical behavior of all specimens with different face sheets-core combination including glass fiber reinforced polymer (GFRP) face sheets and honeycomb core. The results showed a good agreement between the predicted and observed modes.

In addition, other researchers have used the honeycomb-filled othogrid-core sandwich structure with grid panels to improve stiffness and strength of the structure [135]. The load-displacement curve in this study showed a high bending-resistance, which contributes a strong flexural capability, by using a combination design of grid and honeycomb structure. Florence et al. [160] investigated the hybrid FRP honeycomb sandwich panels composites filled with Roahcell, Wheat husk and PUF are experimentally investigated under three point bending test. The simulation results of nonlinear finite elements of honeycomb sandwich panel models were compared to experiments; both simulated and experimental models yield similar results with regard to initial stiffness, peak force, and stiffness degradation [161]. Currently, recent researches were done on triangular honeycombs, and found that they provide better mechanical performance (in-plane stiffness/strength) over various mechanical loading conditions [162]. Several study were done on flexural stiffness properties of GFRP tubes and panels with different types of honeycomb filled as shown in Table 4.

From previous literature, most of the study shows that the flexural behavior of filled-beam and sandwich panel were increased compare to hollow structure. In addition, the used of honeycomb-filled in structure enhancement are become more widespread due to their light weight, high stiffness and good flexural behavior. However, most researchers had used small scale specimens structured, such as in the form of panels in their studies. Therefore, the study on filled-structure behaviors need to be done in actual scale to have more reliable data. Besides that, there is still a lack of study on the flexural behavior of PGFRPC honeycomb-filled structures due to the bending deformation between hollow beams and honeycomb-filled beams. Thus, more experiments and data collection are needed to identify whether the honeycomb core is suitable to be used as a filler for PGFRPC cross-arm structures.

### 6.2. Load-Carrying Capacity Behavior

Recent research has been focusing on different designs of honeycomb-filled structure, good material selection, and parameter optimization to improve load-carrying capacities. Creating hierarchical honeycombs, honeycomb-corrugation hybrids, and grid reinforced honeycombs, and filling the honeycomb holes, are several approaches generally used to improve the strength of the honeycomb structure.

The comparisons of analytical and numerical results from studies have shown an agreement for the load-carrying capacity of the sandwich structure, as studied by Qin et al. [27]. The study also shows that the comparisons of the analytical and numerical results may be overestimated if the effect of local denting is neglected in theoretical analysis. In addition, the filled-structures prevail among composite structures in terms of energy absorption and load-carrying capacity due to their advantages of simple configuration and easy manufacturing [143].

Wang et al. [143,165] investigated honeycomb-filled thin-walled square tube (HFST) structures using experimental and numerical methods, and honeycomb-filled circular tubes (HFCT) using quasi-static compression. It was found that the HFST structures exhibit a more favorable mechanical behavior, with increased energy absorption and load-carrying capacity. While the results for HFCT clearly showed that HFCT structures can greatly increase the load-carrying capacity compared to the hollow ones. In addition, HFCT also exhibits a perfect mechanical response under compression conditions.

**Figure 9:**
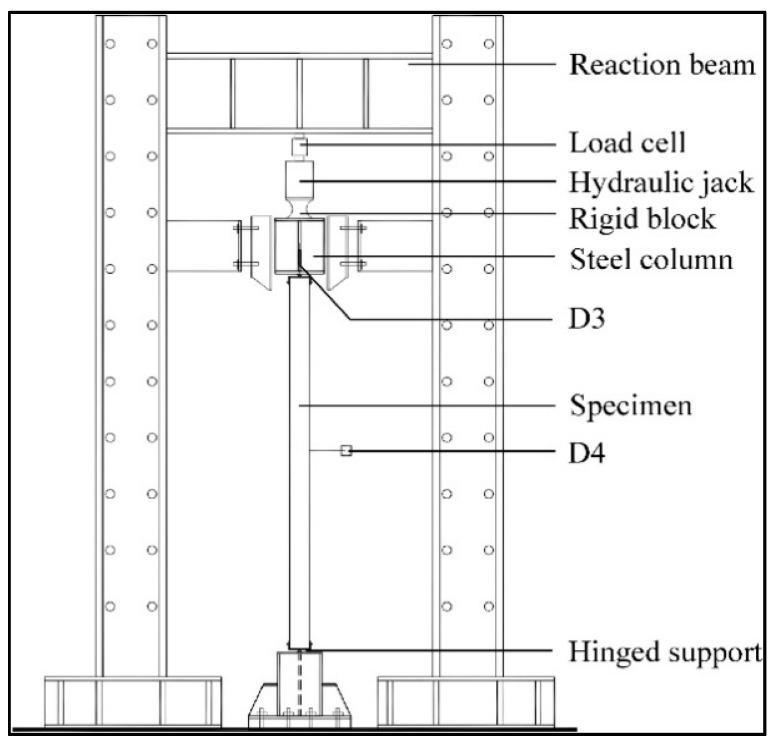
Test setup for residual compressive load-carrying capacity test [168].

To prevent premature failures, aluminum honeycombs of the same areal density with and without foam concrete filling, subjected to quasi-static and dynamic compressions, were experimentally tested; results showed that the load-carrying capacity of the honeycombs increases compared to the corresponding forms of concrete and honeycomb added up separately [166]. The lattice cores, usually with high porosity, possess enough interior interstices for exploring multi-functionalities, such as simultaneous load-carrying and heat dissipation [167]. One of the apparatus used to study the compressive load-carrying capacity test is shown in Figure 9.

The previous paragraph shown the study on honeycomb-filled structure by comparing analytical and numerical results. While others have proved that the load carrying capacity of honeycomb-filled structure exhibit more favorable compare to the hollow ones. However, there are still lack of study on the load carrying capacity with different load types such as axial load, vertical load, concentrated load, and uniform load which are very importance for beam applications. Therefore, more testing and data collection are needed to identify whether the honeycomb-filled structure is good enough to be used as a filler for PGFRPC cross-arm structures.

### 6.3. Creep Behavior

In order to understand the description of loading mechanism for prolonged time, creep behavior needs to be considered. The creep test is conducted to investigate the strength of the structure and material, failure mood, elasticity, and viscoelasticity under constant load in long-term periods [73,169,170,171]. Thus, similar prior studies of honeycomb-filled structures for creep response are highlighted to provide a clear image for any future assessment to be applied to cross-arm structures.

Recent research works have been conducted to study the failure mood and creep properties of the honeycomb foam-filled aluminum tube during a quasi-static compressive test [172]. It was found that the square aluminum tubes filled with both polyurethane foam and aluminum honeycomb have a good creep behavior compared to others. The creep behaviors of composite sandwich beams with glass fiber-reinforced polymer (GFRP) face sheets exhibit linear viscoelastic properties when the load level is less than 40%; creep failure occurs at load levels more than 60% [131]. Furthermore, other researchers have proposed a model which fits the experimental creep curves by using honeycomb-filled sandwich panels in building floors [173]. The findings on creep behavior have also been studied for additive manufacturing and rapid prototyping applications of honeycomb sandwich structures [174]. The result of the study showed a dispersion in strut thickness, while the Plateau border radius only has a mild effect in the creep regime.

In recent past, another researchers have discovered testing facilities to evaluate creep behavior of woods and existing PGFRPC cross arm in coupon strip size and actual scale structure [73]. The creep analysis study can be divided into two category, experimental and numerical analyses study. The experimental works deal with two main methods, which include temperature-based (accelerated) and load-based (conventional) methods. Apart from that, several test rigs simulation design were developed specifically in order to evaluate the creep performance of cross arm in actual outdoor environment (tropical climate condition) [104,128,175].

Limited number of small-scale specimens were tested in previous works. It is highly important for future studies to include large-scale panels made of honeycomb-filled structures to be tested under both conventional and accelerated creep techniques. The creep strain, creep compliance, stress-independent material constant, creep failure, and creep life are interesting topics, especially for cross-arm structures. Hence, previous related studies on creep responses of cross-arm structures are highlighted to provide a clearer picture for any potential evaluation before being commercialized in the energy sector.

### 6.4. Failure Mode Behavior

The combination of internal filler and external structure successively eliminates obsolete methods and puts forth new innovative mechanisms of filling. During compression of the honeycomb structure, deformation behavior can be divided into elastic deformation and plastic deformation [159]. Generally, the magnitude of the elastic deformation of the honeycomb is much smaller than that of the plastic state. However, for honeycomb-filled tube/beam, the failure mode can become complex, including plastic hinges, buckling, indentation, core failure, and shear interaction. Most researchers have proven this by studying simulations and conducting experiments, where a microfracture is initiated in the corner of the top wall in contact with the force applied due to the concentration of stress [62,127]. The cracks will spread to the bottom tube’s surface due to the hinge line. The same phenomenon occurs on filled-honeycomb cell layers, where the honeycomb cell layers squeeze into each other on the upper surface (compression surface) and the cell layers stretch on the bottom surface (tensile surface). Nonetheless, the bending deformation is not sufficient to induce the cells of the aluminum honeycomb to de-bonded from adjacent adhesive layers. As such, de-bonding damages in aluminum honeycomb cell layers are not easy to identify [158].

Hussein et al. investigated failure mood of hollow and honeycomb-filled square CFRP tubes during a compressive load test [172]. By using square hollow aluminum tubes with different lengths and honeycomb-filled conditions, the final deformation mood for honeycomb-filled with gaps between the two sides on the tube was a combination of splaying progressive and transverse shearing failure mode. Another closely related factor of the matching effect on deformation mood is the material’s property between these two key components, filler, and container [167]. By using experimental investigation, Rafea et al. studied the deformation mode of honeycomb-filled square carbon fiber-reinforced plastics (CFRP) and compared it with hollow CFRP tubes; results found a combination of modes of failure [150]. 

**Figure 10:**
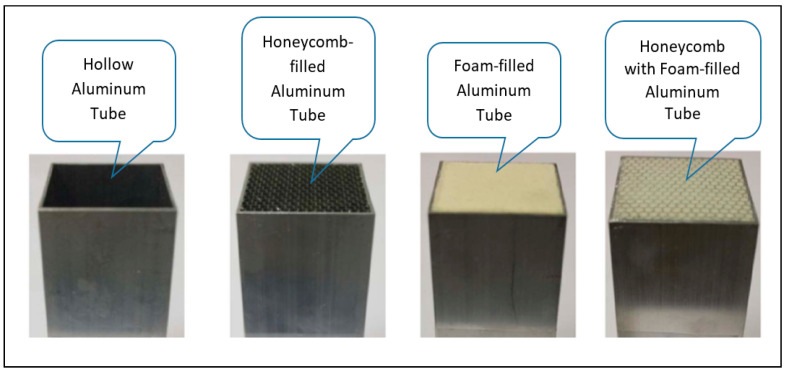
Different innovation of honeycomb filled tube specimens.

Experimental and numerical studies were carried out by Yunwei et al. [176] on the drop weight impact response of the tube-reinforced honeycomb sandwich structure, where the honeycomb holes are filled with metallic tubes. The results showed that the maximum deflections were reduced in the front and back of face-sheets of the globally filled tube-reinforced honeycomb sandwich structure. The finite element model of cylindrical honeycomb with random topological architectures was constructed to study the deformation modes; the results showed that the deformation modes of the structures are significantly affected by the thickness-to-diameter ratio and cell irregularity [177]. Meanwhile, other researchers studied the dynamic impact loading of honeycomb-filled beams as illustrated in Figure 10 [178]. The load-displacement curves of hollow and filled beams under dynamic impact loading by comparing the experimental data with the finite element analysis. It is very clear that the peak forces of tubes filled with the honeycomb structure are increased compared to those of the hollow beams. Furthermore, the crash force efficiency (CFE) also shows an increase over that of hollow beams. 

The deformation behavior of honeycomb-filled structures shows an improvement compared to that of hollow beams due to the combination of internal filler and external structure, successively. Besides that, the matching properties are also once of the considerable properties for deformation mode study. Although most of the previous studies had concentrated on impact and compressive load tests, it is also important to consider the application of cantilever beams and the bending deformation study during load application. Hence, a series of quasi-static mechanical tests and analyses are required to ensure that the fabricated PGFRPC honeycomb-filled composite cross-arm structures fulfil the mechanical property standards as the existing cross-arm in use.

## 7. Conclusions

Of all the aforementioned works from the literature for the application of engineering structures, there have been inadequate ideas and potential value that can overcome PGFRPC cross-arm failures. Presently, these issues are addressed with the notion of temporary strengthening the materials with additional structural members. These measures can only prolong the service life temporarily. In order to provide a better solution, this manuscript presents a brief review on the potential enhancement of PGFRPC cross arm with honeycomb-filled structure.

In the future, the innovative honeycomb-filled structure will still be a hotspot in the field of engineering applications. The general honeycomb-filled concept is proposed for cross arm application compare to tandem and embedded honeycomb concept due to their easy manufacturing and simple configuration. Besides that, the honeycomb-filled structure allow an increase in strength, energy absorbing, flexural behavior, load carrying capacity and creep response. Although, the previous works shows the improvement of sandwich panels and beam which used honeycomb-filled structures compared to hollow structure, however there are still limited information on PGFRPC honeycomb-filled structure performance. Since the use of honeycomb-filled structure concept in enhancement of cross arm structure are new in this field, thus, several studies are needed to be identified and evaluated such as:

1. Improvement of existing manufacturing process of composite structure to have an Economical and highly efficient manufacturing methods of honeycomb-filled PGFRPC cross arm beams.
2. Coupon and actual scale study of honeycomb-filled PGFRPC cross arm on related flexural characteristics behavior, creep responses, load carrying capacity and failure mode behavior.
3. Matching properties of honeycomb-core with PGFRPC beams due to deformation mode behavior.
4. Environmental and global effects of honeycomb-filled PGFRPC beam structure.

All this studies are also opportunities and deserve to be focused on in the future. Once they are overcome with fruitful accomplishments, further significant guidelines will be provided in aid to design the new generation honeycomb-filled PGFRPC based structures.

## Figures and Tables

**Table 1:** Applications of fiber-reinforced polymer composite materials.

Applications	Material	Field Area	Properties	Ref
Fire resistance concrete	Fiber-reinforced inorganic polymer (FRiP) composites	Civil	Improve fire resistance, strengthen concrete structure	[41,42,43]
Concrete beams	Basalt fiber-reinforced polymer (BFRP) composites	Civil	Increase flexural capacity, Improve ductility	[44]
Bridge System-girders, bridge decks, and slab-on-girder bridge systems	Hybrid fiber-reinforced polymer (FRP)-concrete	Civil	Higher durability, less stiffness	[45]
Automobile body parts: Engine hood, dashboard, and storage tank	Natural fiber-reinforced polymer composites	Automobile	Reduce weight, enhance stability and strength, improve in safety features	[39,40,46]
Mechanical Gear pair	Polyoxymethylene (POM) with glass fiber-reinforced polymer composites	Mechanical	Enhance load-carrying capacity	[47,48,49]
Hydraulic cylinder	Carbon fiber-reinforced polymer (CFRP) composites	Mechanical	Weight reduction	[50]
Trunk lid and body stiffeners	Carbon fiber-reinforced polymer (CFRP) composites	Automobile	Higher strength to weight ratio	[51]
Pressure vessel	Fiber-reinforced polymer (FRP) composites	Mechanical	High strength and rigidity, improve corrosion resistance, improved fatigue strength, reduce weight	[52]
Engine hood	Glass fiber-reinforced polymer (GFRP) composites	Automobile	Improve tensile strength and wear resistance properties	[53]
Aircraft interior panels	Natural fiber-reinforced thermoplastic composites	Aerospace	Heat and flame resistance, lightweight, easy recycling	[54,55]
Aircraft parts	Hybrid kenaf/glass fiber-reinforced polymer (KFRP/GFRP) composites	Aerospace	Enhanced rain erosion resistance	[56]
Marine	Hybrid glass-carbon fiber-reinforced polymer composites (GCG_2_C)	Marine	High flexural strength, lowest water absorption tendency	[57]

**Table 2:** Current research progresses of pultruded glass fiber-reinforced polymer composite (PGFRPC) cross arm studies.

Mode of Study	Research	Findings	Ref
Numerical simulation	Effect of laminate properties on cross arm’s failure.	Greater value of young modulus and ultimate strength of a cross arm structure would produce smaller deflection and reduce amount of failure upon multi-axial load condition.	[125]
	Impact of laminate stacking sequence on cross arm’s performance.	Layers proportion with different fiber directions has extraordinary effect on static displacement.	[126]
	Effect of static loading with various configurations on cross arm behaviors.	Addition of bracing system would improve the overall static deformation and stress performance of cross arm	[15]
	Influence of static loadings and sleeve installation on cross arm structure.	The incorporation of sleeve aids to decrease both deformation and stress concentration at the cross arms assembly, which subsequently cause less potential to fatigue failure and higher reliability for the long term service.	[12]
	Modelling of GFRP cross arm using ANSYS and SOLIDWORKS tools.	GFRP cross arm was discovered that it is safe from the failure modes of fiber, matrix, in-plane shear, out-of-plane shear, and delamination under all load conditions which satisfies the ultimate limit state requirements but the concern was on the serviceability limit state which had a deflection of 34 mm.	[127]
Mechanical test rigs development specialized for cross arms	Conceptual design of creep testing rig for full-scale cross arm.	The study implements the TRIZ inventive principles to identify actual test rig problems, morphological chart method to refine the design features, and analytic network process use to select designs. Concept design 5 and 3 were chosen for full-scale and coupon-scale cross arms test rigs.	[103,104]
	Conceptual design of multi-operation outdoor flexural creep test rig		[128]
Experiments	Experimental testing on compressive strength equation for GFRP square tube columns.	Short and intermediate PGFRP beam columns exhibited a significant reduction of capacity due to interaction of rushing, local buckling and global buckling which correspond to each failure.	[129]
	Mechanical evaluation on composite cross arm performance	The axial forces in the main member beams are linearly varying with applied load, whereby the tie member of cross arms which experience axial forces is found to be lesser in magnitude.	[11]

**Table 3:** Energy absorption of hybrid composite with various configurations.

Hybrid	Configuration	Absorbed Energy (J)	Ref
Woven Carbon-Kevlar-glass-fiber	CGC/GCG/KGK	57/59/78	[155]
	GKG/KCK/CKC	90/103/105	
Kevlar-Carbon-glass woven fabrics	KCGKGC/GCKCKG/KGCGCK/GKCCGK/KCGGCK	94.36/95.17/95.01/95.15/95.04/93.16	[156]
Carbon-Kevlar-E-glass fabrics	$\left(GK_{3}C_{4}\right)$_S_	20.35	[157]
	$\left(G_{2}K_{2}C_{4}\right)$_S_	22	
	$\left(G_{4}\;KC_{4}\right)$_S_	22.6	

**Table 4:** Flexural stiffness properties of FRP/GFRP beams/panels with different core-filled structures [31,163,164].

Type	Filler	Flexural Stiffness (Nm^2^)
GFRP Hollow Beam	-	23.8
GFRP Honeycomb-filled tube	Honeycomb	46.49
FRP Honeycomb Foam-filled tube	Honeycomb with Foam-filled	52.94
GFRP wood–filled Beam	Wood	203 G

## Data Availability

The data used to support the findings of this study are included within the article.
